# Supplementary material for: Fast and strong amplifiers of natural selection
Source: Nat Commun. 2021 Jun 29;12:4009. doi: 10.1038/s41467-021-24271-w (PMC8242091; doi:10.1038/s41467-021-24271-w)
Supplement: Supplementary file 1 — Supplementary Information [file 41467_2021_24271_MOESM1_ESM.pdf]

Supplementary Information: Fast and strong amplifiers of natural selection

Josef Tkadlec[1,⋆], Andreas Pavlogiannis[2], Krishnendu Chatterjee[3], and Martin A. Nowak[1]

[1]Department of Mathematics, Harvard University, Cambridge, MA 02138
[2]Department of Computer Science, Aarhus University, Aabogade 34, 8200 Aarhus, Denmark
[3]Institute of Science and Technology Austria, Am Campus 1, 3400 Klosterneuburg, Austria
These authors contributed equally: Josef Tkadlec and Andreas Pavlogiannis
⋆To whom correspondence should be addressed: josef.tkadlec@gmail.com

Contents

1 Supplementary Note 1: Model **2**
1.1 Evolutionary graph theory . . . 2
1.2 Results for large N . . . 3
1.3 Selection reactors . . . 4
1.4 Mathematical tools . . . 4

2 Supplementary Note 2: Results **5**
2.1 Proof idea for Theorem 1 . . . 6
2.2 Proof idea for Theorem 2 . . . 6

3 Supplementary Note 3: Proofs **7**
3.1 No superamplifiers are as fast as the complete graph . . . 7
3.2 Selection reactors are superamplifiers . . . 11
3.3 Selection reactors are fast superamplifiers . . . 14

This is a supplementary note to the manuscript Fast and strong amplifiers of natural selection. The organization of the text is as follows: In Supplementary Note 1 we introduce the necessary terminology and notation, together with the tools we will be using in the proofs. In Supplementary Note 2 we formally state our mathematical results and provide a high level overview of the ideas behind our proofs. In Supplementary Note 3 we then provide those formal proofs.

1 Supplementary Note 1: Model

1.1 Evolutionary graph theory

Here we formally define Moran Birth-death process on a population structure, the key quantities of fixation probability and timescale of fixation, and the notions of amplifiers and strong amplifiers.

Moran process on a population structure. The population structure is described by a connected graph (network) $G = (V, E)$, where V is a set of $|V| = N$ nodes and E is a set of edges, possibly including self-loops, where each edge uv is assigned a weight w_{uv}. The nodes of G represent sites, each site hosting one individual. The individuals are of two types: *residents* with fitness normalized to 1, and *mutants* with (relative) fitness advantage $r > 1$. The set of nodes occupied by mutants is called a *configuration.* Given a configuration $\mathcal{S}$ we denote by $\mathsf{F}(\mathcal{S})$ the total fitness of the population. The edges of G represent connections between adjacent sites, marking where an individual could place its offspring, and the weights represent the strengths of those interactions. The *Moran Birth-death process* is a stochastic (random) process that acts in discrete time steps until either all nodes are occupied by mutants (*fixation* occured) or by residents (*extinction* occurred): At each time step, first ("Birth") an individual is selected randomly proportionally to its fitness and it produces a copy of itself. Denote the corresponding site by u. Second ("death"), a site v adjacent to u is selected randomly proportionally to the weight w_{uv} of the edge uv, and the individual at site v gets replaced by a copy of the individual at site u.

Fixation probability, Extinction probability, Fixation time. Given a graph G with N nodes, a mutant advantage $r > 1$ and a single node v, we denote by $\mathrm{fp}(G, r, v)$ the *fixation probability* of the Moran Birth-death process, starting with a configuration containing a single mutant with fitness r occupying node v, against a background population of residents with fitness normalized to 1. Similarly, we denote by $\mathrm{ep}(G, r, v) = 1 - \mathrm{fp}(G, r, v)$ the *extinction probability.*

Concerning the duration of the process, we denote by $\mathrm{FT}(G, r, v)$ the (unconditional) *fixation time*, that is, the (expected) number of steps the process makes until either the mutants or the residents fixate, divided by the population size N. (This quantity, without rescaling by the factor of N, is sometimes also called the *absorption time.*) Note that the fixation time counts all the steps of the process, including those where the set of nodes occupied by mutants might not have changed due to an offspring replacing an individual of the same type. The argument for rescaling the fixation time by the population size N is that Moran process can be viewed as a discretization of a continuous-time Markov chain where a node with fitness F reproduces after a time given by an exponentially distributed random variable with mean $1/\mathsf{F}$ (by listing only the timepoints in which a reproduction event has happened). In this continuous viewpoint, a population with double the size undergoes twice as many reproduction events per unit time. Thus we prefer to measure the fixation time in generations rather than in individual steps.

Finally, for completeness we briefly mention another quantity that is sometimes used to measure time. We denote by $\mathrm{CFT}(G, r, v)$ the *conditional fixation time*, that is, the (expected) number of steps the process makes until the mutants fixate, conditioning on the fact they do, divided by the population size N. In other words, when computing the conditional fixation time, we average over only those evolutionary trajectories that eventually reach fixation of the mutant. We note that conditional fixation time is typically longer than the (unconditional) fixation time, since the trajectories leading to fixation are typically longer than those leading to extinction. Similarly, for strong amplifiers (defined below), the fixation time and the conditional fixation time are typically asymptotically equivalent, since the fixation event has probability 1.

Uniform and temperature initialization. Averaging over the $|V| = N$ possible locations of the initial mutant, we denote by

$$\mathrm{fp}(G, r) = \frac{1}{N} \sum_{v \in V} \mathrm{fp}(G, r, v)$$

the fixation probability under *uniform initialization.* This is meaningful under the assumption that mutations arise spontaneously, with the same rate at each node. If one assumes that mutations arise during reproduction, one prefers

to study the weighted average

$$\mathrm{fp}^{\mathcal{T}}(G,r) = \frac{1}{N}\sum_{v\in V}(\mathcal{L}(v)+\mathcal{T}(v))\cdot \mathrm{fp}(G,r,v),$$

where $\mathcal{L}(v) = \frac{w_{vv}}{\sum_{v'} w_{vv'}}$ is the probability that when a node v reproduces it self-loops, and $\mathcal{T}(v) = \sum_{u\in V\setminus\{v\}} \frac{w_{uv}}{\sum_{v'} w_{uv'}}$ is the *temperature* of node v. Intuitively, the temperature of a node v counts how many times per generation is v replaced by its neighbors, on average, when all individuals are residents. Therefore the temperature initialization can be thought of as follows: Consider a uniform population of residents, sample a random reproduction event, and then place a mutant on the node being replaced. The extinction probability $\mathrm{ep}(G,r)$, $\mathrm{ep}^{\mathcal{T}}(G,r)$ and the fixation time $\mathrm{FT}(G,r)$, $\mathrm{FT}^{\mathcal{T}}(G,r)$ under uniform and temperature initialization are defined analogously.

Complete graph K_N, amplifiers. The well-mixed ("unstructured") population consisting of N individuals is modeled by a complete graph K_N. It is known [1, 2, 3] that for any fixed $r>1$ we have

$$\mathrm{fp}(K_N,r) = \frac{1-1/r}{1-1/r^N} \to_{N\to\infty} 1-1/r \qquad \text{and} \qquad \mathrm{FT}(K_N,r) = (1+1/r)\cdot\log N + o(\log N)$$

as $N\to\infty$. This gives a natural baseline to which one can compare the other population structures. In particular, given $r>1$, a population structure A_N with N nodes is called an *r-amplifier* if it increases the fixation probability as compared to the complete graph of the same size (i.e. $\mathrm{fp}(A_N,r) > \mathrm{fp}(K_N,r)$). A population structure that is an r-amplifier for all $r>1$ is called an *amplifier.* (We note that other authors sometimes further require that a graph A_N satisfies $\mathrm{fp}(A_N,r) < \mathrm{fp}(K_N,r)$ for all $r<1$ to be called an amplifier.)

1.2 Results for large N

Superamplfication and timescale of fixation concern the behavior of population structures in the limit of a large population size $N\to\infty$. First we recall the standard mathematical notation for asymptotic results.

Notation for asymptotic results. In the limit $N\to\infty$, we consider the standard mathematical notation of $o(\cdot)$, $\mathcal{O}(\cdot)$, $\Omega(\cdot)$, $\omega(\cdot)$, and $\Theta(\cdot)$ that denote asymptotically strictly less than, less than or equal to, greater than or qual to, strictly greater than, and asymptotically equal to (up to a constant factor), respectively. Finally, we use the approximation sign $x\approx y$ to state that $y = x+o(x)$. As four examples, we will write:

- $\frac{1}{N} = o(1)$ since $\lim_{N\to\infty}\frac{1}{N}/1 = 0$;
- $\sqrt{N} = \omega(\log N)$ since $\lim_{N\to\infty}(\sqrt{N})/(\log N) = \infty$;
- $\frac{1}{2}N(N+1) = \Theta(N^2)$ since $\lim_{N\to\infty}\frac{1}{2}N(N+1)/(N^2) = \frac{1}{2}$ is a non-zero constant; and
- $\frac{1}{2}N(N+1) = \frac{1}{2}N^2 + \frac{1}{2}N \approx \frac{1}{2}N^2$, since $\frac{1}{2}N = o(\frac{1}{2}N^2)$.

For detailed treatment see [4, Section 1.3].

We say that an event happens *with high probability* if the probability p_N that the event happens tends to 1 as N tends to infinity (equivalently, if $1-p_N = o(1)$).

Strong amplifiers and Timescale of fixation. In order to compare the behavior of population structures for large N, we consider sequences $\{G_N\}_{N=1}^{\infty}$ of graphs of increasing population size.

Regarding probability, such a sequence $\{G_N\}_{N=1}^{\infty}$ is called a *strong amplifier* (also known as a *superamplifier*) if, for any fixed $r>1$, it satisfies $\mathrm{fp}(G_N,r)\to_{N\to\infty} 1$. Strong amplification is, in a sense, the strongest possible form of amplification – an arbitrarily minute (but fixed) fitness advantage $r>1$ guarantees that a single mutant with that advantage will fixate with high probability, as the population size N grows large.

Regarding the duration of the process, we define the timescale of fixation by focusing on how the fixation time dependends on N and ignoring both the lower order terms and the possible constants that depend on r but not on N. For instance, for Complete graphs K_N we have $\mathrm{FT}(G_N,r) = (1+1/r)\cdot\log N + o(\log N)$, hence for any fixed $r>1$ we can write $\mathrm{FT}(G_N,r) = \Theta(\log N)$. Formally, given a sequence $\{G_N\}_{N=1}^{\infty}$ and $r>1$ the *timescale of fixation* is a function $\mathrm{T}(G_N,r)$ such that $\mathrm{FT}(G_N,r) = \Theta(\mathrm{T}(G_N,r))$. If the same function $\mathrm{T}(G_N,r)$ applies for all $r>1$, as is the case for instance for the Complete graphs, we omit the parameter r and write $\mathrm{T}(G_N)$.

1.3 Selection reactors

In this section we introduce Selection reactors. First we describe their underlying (unweighted) graph structure.

Unweighted selection reactor. Given integers N, n the unweighted selection reactor $\mathrm{USR}_N(n)$ is an unweighted graph with N nodes such that:

1. There are n *hub* nodes and $N-n$ *leaf* nodes.
2. Every two hub nodes are connected, every hub node is connected to every leaf node, and every leaf node has a self-loop.

Firing in and out. Due to the symmetry of selection reactors, any step of the process that changes the mutant configuration is one of the following four types:

1. A hub node replaces a leaf node: We say the hub node *fires out*.
2. A hub node replaces another hub node: We say the hub node does not fire out (or *stays*).
3. A leaf node replaces a hub node: We say the leaf *fires in*.
4. A leaf node replaces itself due to a self loop: We say the leaf does not fire in (or *loops*).

Moreover, we say that a fire-in event is a *resident fire-in* when the individual reproducing is a resident (and similarly for mutant and/or fire-out events).

Selection reactors. Now we can formally define (weighted) Selection reactors. Given integers N, n and two real numbers $p_{\text{in}}, p_{\text{out}} \in (0,1)$, the selection reactor $\mathrm{SR}_N(n, p_{\text{in}}, p_{\text{out}})$ is a weighted graph with N nodes such that:

1. There are n *hub* nodes and $N-n$ *leaf* nodes.
2. Every two hub nodes are connected by an edge with weight w_h, every leaf node has a self-loop with weight w_l, and every hub node is connected to every leaf node by an edge with weight $\mathsf{w}_a = 1$. The numbers w_h and w_l are chosen such that:
   (a) For any leaf node, once it is selected for reproduction, it fires in the hub (as opposed to self-looping) with probability p_{in}. In other words, we expect to see $(N-n)\cdot p_{\text{in}}$ fire-in events per generation.
   (b) For any hub node, once it is selected for reproduction, it fires out to some leaf (as opposed to replacing another hub node) with probability p_{out}. In other words, we expect to see $n \cdot p_{\text{out}}$ fire-out events per generation.

It is readily checked that the properties 2a and 2b are satisfied by assigning weight $\mathsf{w}_h = (N-n)(1-p_{\text{out}})/((n-1)p_{\text{out}})$ to each edge within the hub, and by assigning weight $\mathsf{w}_l = n \cdot (1-p_{\text{in}})/p_{\text{in}}$ to each self-loop, respectively.

1.4 Mathematical tools

Here we introduce the mathematical notions and tools required for our proofs.

Martingales. Note that Moran process on a population structure $G = (V, E)$ can be represented as an absorbing Markov chain, whose states correspond to all the possible configurations $S \subseteq 2^V$ (i.e. subsets of nodes occupied by mutants) and whose transition probabilities can be expressed in terms of the weights of the edges. We analyze such Markov chains using the machinery of martingales: Intuitively, a martingale is a "potential function" $\phi\colon 2^V \to \mathbb{R}$ that assigns a real number to every configuration $S \in 2^V$ such that, in expectation, the value of the potential does not change in one step of the Moran process. Formally, we consider the sequence $\{\mathcal{S}_t\}_{t=0}^{\infty}$ of random variables where $\mathcal{S}_0$ is the (possibly random) initial configuration of the process and $\mathcal{S}_t$ is the configuration after t steps. We say that a function ϕ is a *martingale* if

$$\mathbb{E}[\phi(\mathcal{S}_{t+1}) \mid \mathcal{S}_t] = \phi(\mathcal{S}_t)$$

Similarly, we say that a function ψ is a *sub-martingale* if, in expectation, it does not decrease, that is if

$$\mathbb{E}[\psi(\mathcal{S}_{t+1}) \mid \mathcal{S}_t] \geq \psi(\mathcal{S}_t)$$

Example martingale. As an example, consider Moran process running on a complete graph $G = K_N$ and any $r \geq 1$. Given any configuration S, let p_S^+ (resp. p_S^-) be the probability that in the next step we gain (resp. lose) one mutant. Let $\mathsf{F}(S) = N + (r-1) \cdot |S|$ be the total fitness of the population. Then we have

$$p_S^+ = \frac{r \cdot |S|}{\mathsf{F}(S)} \cdot \frac{N - |S|}{N-1} \quad \text{and} \quad p_S^- = \frac{N-|S|}{\mathsf{F}(S)} \cdot \frac{|S|}{N-1},$$

hence $p_S^+ = r \cdot p_S^-$.

Now consider a function ϕ_c that counts the number of mutants in the current configuration. Formally ϕ_c is defined as

$$\phi_c(\mathcal{S}_t) = |\mathcal{S}_t|.$$

We claim that ϕ_c is a martingale when $r = 1$ and a sub-martingale when $r > 1$: Indeed,

$$\mathbb{E}[\phi_c(\mathcal{S}_{t+1}) \mid \mathcal{S}_t] = \phi_c(\mathcal{S}_t) + p_{\mathcal{S}_t}^+ \cdot (+1) + p_{\mathcal{S}_t}^- \cdot (-1) = \phi_c(\mathcal{S}_t) + (r-1) \cdot p_{\mathcal{S}_t}^-,$$

where the last term is 0 when $r = 1$ and non-negative when $r > 1$.

Specific mathematical tools. We make use of the following standard results.

Proposition 1 (Azuma-Hoeffding inequality)**.** Suppose $\{\mathcal{S}_t\}_{t=0}^{\infty}$ is a sub-martingale and there exists $d \geq 0$ such that $|\mathcal{S}_{k+1} - \mathcal{S}_k| \leq d$ for any $k \geq 0$. Then for any $t \geq 0$ and any real $\varepsilon > 0$ we have

$$\mathbb{P}[\mathcal{S}_t - \mathcal{S}_0 \leq -\varepsilon] \leq \exp\left(\frac{-\varepsilon^2}{2t \cdot d^2}\right).$$

Proposition 2 (Markov's inequality)**.** Let X be nonnegative random variable with expectation $\mu = \mathbb{E}[X]$ and let $\lambda > 0$ be a real number. Then

$$\mathbb{P}[X > \lambda \cdot \mu] \leq \frac{1}{\lambda}.$$

Proposition 3 (Limit for $1/e$)**.** Suppose $f(N) = o(N)$. Then $\lim_{N\to\infty}(1 - 1/N)^{f(N)} = 1$.

Proposition 4 (Asymptotics of the harmonic series)**.** For any $N \geq 1$ we have $\log N \leq \sum_{i=1}^{N} 1/i \leq \log N + 1$.

2 Supplementary Note 2: Results

Here we restate our two main results from the main text and present the high level ideas behind their proofs.

Conceptually, our first main result shows that in order to achieve strong amplification, some asymptotic slowdown is necessary. In other words, there are no strong amplifiers that would be only constant factor slower than complete graphs.

Theorem 1. *For any strong amplifier $\{G_N\}_{N=1}^{\infty}$ there exists an increasing unbounded function $\beta(N)$ such that*

$$\mathrm{T}(G_N) \geq \log N \cdot \beta(N).$$

To complement this, our second main result shows that strong amplification can be achieved with an arbitrarily small slowdown as compared to the complete graph. The slowdown is given by a function $\beta(N)$ which can increase arbitrarily slowly as long as it is unbounded.

Theorem 2. *For any increasing unbounded function $\beta(N)$ there exists a strong amplifier $\{G_N\}_{N=1}^{\infty}$ such that*

$$\mathrm{T}(G_N) \leq \log N \cdot \beta(N).$$

In fact, our two main results (Theorem 1 and Theorem 2) can be viewed as immediate corollaries of two slightly more general theorems (Theorem A and Theorem B, respectively). Next we state those two more general theorems and we provide the high level ideas behind their proofs. The full formal proofs appear in Section 3.

2.1 Proof idea for Theorem 1

Theorem 1 follows directly from Theorem A which, for any fixed $r > 1$ and any amplifier (not necessarily a strong amplifier), gives a lower bound on fixation time that is inversely proportional to the extinction probability.

Theorem A. *Fix $r > 1$.*

1. *If G is an r-amplifier under uniform initialization then*

$$\mathrm{FT}(G,r) \geq \frac{(r-1)^2}{r^4(r+1)} \cdot \frac{1}{\mathrm{ep}(G,r)} \cdot \log\left(N \cdot \frac{r-1}{r+1} - 1\right) = \Omega\left(\frac{1}{\mathrm{ep}(G,r)} \cdot \log N\right).$$

2. *If G is an r-amplifier under temperature initialization then*

$$\mathrm{FT}^{\mathcal{T}}(G,r) \geq \frac{(r-1)^2}{r^4(r+1)} \cdot \frac{1}{\mathrm{ep}^{\mathcal{T}}(G,r)} \cdot \log\left(N \cdot \frac{(r-1)^2}{r(r+1)^2} - 1\right) = \Omega\left(\frac{1}{\mathrm{ep}^{\mathcal{T}}(G,r)} \cdot \log N\right).$$

Note that this indeed immediately implies Theorem 1: When $\{G_N\}_{N=1}^{\infty}$ is a strong amplifier, then for any fixed $r > 1$ the extinction probability tends to zero in the limit $N \to \infty$, thus $\mathrm{FT}(G_N, r) = \Omega\left(\frac{1}{\mathrm{ep}(G,r)} \cdot N \log N\right) = \omega(N \log N)$ and the slowdown as compared to the complete graph is asymptotically non-negligible.

Proof Idea. The proof of Theorem A rests on two ideas. The first idea is that the fixation time on any graph G can be bounded from below in terms of temperatures of its nodes (see Lemma 1) by noting that, on any trajectory to fixation, each node (except for the node where the initial mutant appeared) has to be replaced at least once by one of its neighbors: In particular, if many nodes have a relatively low temperature ("cold nodes") then the fixation time is relatively long since we must wait until each of the cold nodes is replaced at least once (this corresponds to a coupon-collector-like process with many rare coupons).

The second idea is that when $\mathrm{fp}(G,r)$ is relatively high then there exist many initial nodes u for which $\mathrm{ep}(G,r,u)$ is relatively low. However, when $\mathrm{ep}(G,r,u)$ is low then $\mathcal{T}(u)$ has to be low too – otherwise u would be likely to be replaced by one of its neighbors before reproducing even once. Thus any graph G with high fixation probability inevitably contains many cold nodes and the general bound given by Lemma 1 becomes particularly strong for strong amplifiers.

2.2 Proof idea for Theorem 2

Theorem 2 immediately follows from Theorem B which states that selection reactors with suitably tuned parameters are strong amplifiers whose fixation time is asymptotically arbitrarily close to the fixation time of the complete graphs. We believe that the proof of Theorem B can be modified to apply to sparse incubators introduced in [5] but for convenience in what follows we focus solely on Selection Reactors. Specifically, for any function $\alpha\colon \mathbb{N} \to \mathbb{N}$ that is both $o(N^{1/2})$ and $\omega(1)$, and for any integer N, let

$$\mathrm{SR}_N^{\alpha} = \mathrm{SR}_N(n = \lfloor N/\alpha(N) \rfloor, p_{\mathrm{in}} = 1/\alpha^2(N), p_{\mathrm{out}} = 1/\alpha^2(N))$$

be the selection reactor with $n = \lfloor N/\alpha_N \rfloor$ hub nodes, $\ell = N - n \approx N$ leaf nodes in the periphery, and with fire-in and fire-out rates both equal to $p_{\mathrm{in}} = p_{\mathrm{out}} = 1/\alpha^2(N)$. With this notation we have the following theorem.

Theorem B (Reactors are fast strong amplifiers)**.** *For any function $\alpha\colon \mathbb{N} \to \mathbb{R}$ that is both $o(N^{1/2})$ and $\omega(1)$, and for any fixed $r > 1$, the selection reactors $\{\mathrm{SR}^{\alpha}\}_{N=1}^{\infty}$ satisfy the following, under both uniform and temperature initialization:*

1. *(strong amplifiers)* $\mathrm{fp}(\mathrm{SR}_N^{\alpha}, r) \to_{N\to\infty} 1$.
2. *(fast)* $\mathrm{FT}(\mathrm{SR}_N^{\alpha}, r) = \mathcal{O}(\log N \cdot \alpha^5(N))$.

Note that this indeed immediately implies Theorem 2 as one can take $\alpha(N) = \sqrt[5]{\beta(N)}$.

Before we present the high level idea behind the proof of Theorem B, we need to introduce more notions. Recall that once a leaf node is selected for reproduction, it fires in the hub with probability p_{in}. Similarly, a hub node, once selected, fires out with probability p_{out}. We will make extensive use of a key parameter

$$h = \frac{p_{\mathrm{in}}/n}{p_{\mathrm{out}}/\ell} = \frac{\ell \cdot p_{\mathrm{in}}}{n \cdot p_{\mathrm{out}}}$$

that characterizes the bias towards the hub along any fixed edge that connects a leaf node with a hub node. Our proofs rely on the fact that $h = \omega(1)$ and on several other properties, namely: $n = \omega(1)$, $n = o(N)$, $p_{\text{out}} = o(1)$, $Np_{\text{in}} = o(n)$, $h = o(n)$. For the parameters stated in Theorem B, we have $\ell = \Theta(N)$ and $p_{\text{in}} = p_{\text{out}} = 1/\alpha^2(N)$ and thus $h = \Theta(\alpha(N))$ and all the properties are satisfied.

Proof idea for probability. Here we sketch the intuition behind our proof that Selection reactors from Theorem B are strong amplifiers. The argument proceeds in five steps. We show that:

1. Whenever the hub contains $j \in [1, n/2]$ mutants, the size of the mutant population in the hub is more likely to increase than to decrease, see Lemma 4. This is because the hub by itself is biased towards gaining mutants (due to $r > 1$), and the leaves do not change this as they interact with the hub only rarely.
2. With high probability, the initial mutant appears at a leaf (for both uniform and temperature initialization), see Lemma 5. This is a simple computation.
3. With high probability, that initial mutant repeatedly fires in the hub before being eliminated and, by the result of step 1, this establishes a mutant subpopulation in the hub that has a superconstant size, see Lemma 6.
4. By Item 1 that subpopulation of mutants in the hub grows to size $n/2$, with high probability, see Lemma 7. This is because the hub is biased towards gaining mutants.
5. Once there are $j \geq n/2$ mutants in the hub, fixation on the whole graph occurs with high probability, see Lemma 8. This is because, at this point, the mutants have established such a large subpopulation that an extinction is extremely unlikely.

Proof idea for time. Here we sketch the intuition behind our proof that Selection reactors from Theorem B are *fast* strong amplifiers. Recall that a configuration S is the subset of nodes that are occupied by mutants. We say that a step of the process is *active* if it changes the configuration (due to a mutant replacing a resident or vice versa). The idea is to decompose the process into the *active process* that tracks the active steps and the *waiting process* that tracks the steps in which the configuration S stays the same (either due to a node self-looping or due to a node replacing a neighbor of the same type). The argument then proceeds in four steps:

1. We define a certain potential function ψ that assigns a non-negative integer to each configuration and satisfies $\psi(S) = \mathcal{O}(\ell \cdot h)$ for any configuration S.
2. For the active process, we argue that, in expectation, the value of the potential function increases by at least a constant in each active step (and always by at most $\mathcal{O}(h)$ in one step). This then implies that, in expectation, the active process attains each potential value k only a few times (namely $R_k = \mathcal{O}(h^2)$ times), see Lemma 9 and Lemma 10.
3. For the waiting process, we establish an upper bound on the number W_k of (waiting) steps that the process makes until an active step occurs, when the current configuration S is assigned potential value $k = \psi(S)$, see Lemma 11 and Lemma 12.
4. By linearity of expectation, this implies that, in expectation, the process makes in total at most $\sum_k R_k \cdot W_k = \mathcal{O}(h^2) \cdot \sum_k W_k$ steps. Summing up our upper bounds for W_k from previous item gives the result.

3 Supplementary Note 3: Proofs

In the next two subsections we present the formal proofs of our two main theorems.

3.1 No superamplifiers are as fast as the complete graph

The goal of this section is to prove Theorem A.

First, we present a lower bound on the fixation time for an arbitrary graph G in terms of the temperatures of its nodes. The bound is based on the fact that, on any trajectory to fixation, each node has to be replaced by one of its neighbors at least once. This reduces to a coupon collector process where nodes with lower temperature correspond to coupons that are more rare. Flajolet et al [6] found a closed form expression for the expected number of steps of such a coupon collector process, for any given list of temperatures. Here we present a simplified (weaker) bound that takes into account only those nodes whose temperature is lower than a given threshold ("cold" nodes). The proof idea is similar to the proof of Theorem 1 in [3].

Lemma 1. Let G be a graph, u a node of G and $r > 1$. Suppose that at least k nodes of G different from u have temperature at most $t^\star$ each. Then

$$\mathrm{FT}(G,r,u) \geq \mathrm{fp}(G,r,u) \cdot \frac{\log k}{r \cdot t^\star}.$$

Proof. Consider a modified Moran process M' that is identical with the standard Moran process with a mutant initialized at u, except that whenever the mutants go extinct we instead again initialize a single mutant at u and continue the process. Clearly, the modified process M' always terminates with the mutants fixating. We claim that the expected fixation time is given by $\mathrm{FT}'(G,r,u) = \frac{1}{\mathrm{fp}(G,r,u)} \cdot \mathrm{FT}(G,r,u)$: Indeed, for M denote by $p = \mathrm{fp}(G,r,u)$ the fixation probability and by $E = \mathrm{ET}(G,r,u)$, $C = \mathrm{CFT}(G,r,u)$, $T = \mathrm{FT}(G,r,u)$, the (expected) extinction time, conditional fixation time, and the fixation time, respectively. Then we have $T = p \cdot C + (1-p) \cdot E$. Denoting by $T' = \mathrm{FT}'(G,r,u)$ the fixation time of M', by linearity of expectation we have

$$T' = p \cdot C + (1-p) \cdot (E + T'),$$

since the first mutant either fixates, in which case M' does C steps on average and then terminates (probability p), or goes extinct, in which case M' does E steps on average and then restarts (probability $1-p$). This now rewrites as the desired

$$T' = \frac{p \cdot C + (1-p) \cdot E}{1 - (1-p)} = \frac{T}{p}.$$

Let $C \subseteq V$ be a set of k nodes with temperature at most $t^\star$ each (the "cold" nodes). Since the process M' eventually reaches fixation, for each $i = 0, \ldots, k-1$ it transitions from some configuration with i mutants in C to some configuration with $i+1$ mutants in C at least once. Any time the process is in a configuration with i mutants in C, the probability p_i^+ that it successfully gains another mutant in C satisfies $p_i^+ \leq \frac{r}{N} \cdot (k-i) \cdot t^\star$, because each of the remaining $k-i$ resident nodes in C has temperature at most $\mathcal{T}(v) \leq t^\star$ and thus it is replaced with probability at most $\mathrm{replace}(v) \leq \frac{r}{N} t^\star$. Therefore the expected number of generations spent in such configurations is $t_i \geq \frac{1}{N} \cdot 1/p_i^+ \geq 1/(r(k-i) \cdot t^\star)$. By linearity of expectation, summing over $i = 0, \ldots, k-1$ we get

$$\begin{aligned}\mathrm{FT}(G,r,u) &= \mathrm{fp}(G,r,u) \cdot \mathrm{FT}'(G,r,u) \geq \mathrm{fp}(G,r,u) \cdot \sum_{i=0}^{k-1} t_i \geq \mathrm{fp}(G,r,u) \cdot \sum_{i=0}^{k-1} \frac{1}{r(k-i) \cdot t^\star} \\ &\geq \mathrm{fp}(G,r,u) \cdot \frac{\log k}{r \cdot t^\star}. \end{aligned}$$

□

Remark. We remark that Lemma 1 yields lower bounds on fixation time for various specific graph families and initialization schemes. For instance, star graphs S_N contain $N-1 = \Theta(N)$ nodes of temperature $1/(N-1) = \Theta(1/N)$ each, and therefore for each node u we have

$$\mathrm{FT}(S_N,r,u) \geq \mathrm{fp}(S_N,r,u) \cdot \frac{\log(N-2)}{r \cdot \frac{1}{N-1}} = \mathrm{fp}(S_N,r,u) \cdot \Omega(N \log N).$$

For uniform initialization we have $\mathrm{fp}(S_N,r) \to 1 - 1/r^2 = \Theta(1)$, thus averaging over the initial nodes we obtain $\mathrm{FT}(S_N,r) = \Omega(N \log N)$ which is asymptotically tight [3]. Similarly, dense incubator D_N (see [5] for their definition) are superamplifiers that contain $\Theta(N)$ nodes of temperature $\Theta(N^{-2/3})$ each, thus $\mathrm{FT}(D_N,r) = \Omega(N^{2/3} \log N)$.

In order to prove Theorem A using Lemma 1 we aim to show that any superamplifier has many nodes with low temperature. To that end, we use two lemmas. The first one (Lemma 2) allows us to look for nodes u with small extinction probability $\mathrm{ep}(G,r,u)$ (instead of nodes with low temperature $\mathcal{T}(u)$). The second one (Lemma 3) states that for any graph G that amplifies (under either uniform or temperature initialization), a constant portion of its nodes have a small extinction probability $\mathrm{ep}(G,r,u)$.

Lemma 2. Fix $r > 1$. Let G be a graph and u its node. Denote by $\mathrm{ep}_u = \mathrm{ep}(G,r,u)$ the extinction probability of a single mutant starting at node u. Then

$$\mathcal{T}(u) \leq \frac{r \cdot \mathrm{ep}_u \cdot (1 - \mathcal{L}(u))}{1 - \mathrm{ep}_u} \leq \frac{r \cdot \mathrm{ep}_u}{1 - \mathrm{ep}_u}.$$

Proof. Assume that the configuration of the Moran process at some step t is $\mathcal{S}_t = \{u\}$. Let p (resp., q) be the probability that $|\mathcal{S}_{t+1}| = 2$ (resp., $|\mathcal{S}_{t+1}| = 0$). The event $|\mathcal{S}_{t+1}| = 2$ occurs when u is selected for reproduction and it does not self-loop. The event $|\mathcal{S}_{t+1}| = 0$ occurs when u is replaced by one of its neighbors. Hence

$$p = \frac{r}{\mathsf{F}(\mathcal{S}_t)} \cdot (1 - \mathcal{L}(u)) \qquad \text{and} \qquad q = \sum_{v \neq u} \frac{1}{\mathsf{F}(\mathcal{S}_t)} \cdot \frac{w_{vu}}{\sum_{v'} w_{vv'}} = \frac{1}{\mathsf{F}(\mathcal{S}_t)} \cdot \mathcal{T}(u).$$

Note that if $|\mathcal{S}_{t+1}| \neq 2$ and $|\mathcal{S}_{t+1}| \neq 0$, then we have $\mathcal{S}_{t+1} = \mathcal{S}_t = \{u\}$, therefore the extinction probability from u is lower-bounded by

$$\mathrm{ep}_u \geq \frac{q}{q+p} \geq \frac{\mathcal{T}(u)}{\mathcal{T}(u) + r\cdot(1-\mathcal{L}(u))}.$$

Clearing the denominators and using $1-\mathcal{L}(u) \leq 1$ we get the desired bounds. □

Lemma 3. Fix $r>1$ and set $c_r = \frac{1}{2}(r+1) > 1$. Consider a graph G.

1. (Uniform initialization) Let $S = \{u \in V\colon\ \mathrm{ep}(G,r,u) \leq c_r \cdot \mathrm{ep}(G,r)\}$. Then $|S| \geq N \cdot \frac{r-1}{r+1}$.
2. (Temperature initialization) Similarly, let $S^{\mathcal{T}} = \{u \in V\colon\ \mathrm{ep}(G,r,u) \leq c_r \cdot \mathrm{ep}^{\mathcal{T}}(G,r)\}$. If G is an r-amplifier under temperature initialization then $|S^{\mathcal{T}}| \geq N \cdot \frac{(r-1)^2}{r(r+1)^2}$.

Proof. We prove the two claims independently (but along the same lines).

1. Let X be a random variable that denotes the extinction probability of a node of V chosen uniformly at random. Note that X is non-negative, and

$$\mathbb{E}[X] = \sum_{u\in V} \frac{1}{N}\cdot \mathrm{ep}(G,r,u) = \mathrm{ep}(G,r).$$

By Markov's inequality, we have

$$\mathbb{P}[X > c_r \cdot \mathrm{ep}(G,r)] \leq \frac{\mathbb{E}[X]}{c_r\cdot \mathrm{ep}(G,r)} = \frac{1}{c_r}.$$

Finally, note that

$$1-\frac{1}{c_r} \leq \mathbb{P}[X \leq c_r\cdot \mathrm{ep}(G,r)] = \frac{|S|}{|V|} = \frac{|S|}{N}$$

and thus $|S| \geq N\cdot(1-1/c_r) = N\cdot \frac{r-1}{r+1}$ as claimed.

2. Likewise, let Y be a random variable that denotes the extinction probability of a node of V chosen according to the temperature initialization. Proceeding as before,

$$\mathbb{E}[Y] = \frac{1}{N}\sum_{u\in V}(\mathcal{T}(u)+\mathcal{L}(u))\cdot \mathrm{ep}(G,r,u) = \mathrm{ep}^{\mathcal{T}}(G,r)$$

and since Y is non-negative, by Markov's inequality, we have

$$\mathbb{P}[Y > c_r\cdot \mathrm{ep}^{\mathcal{T}}(G,r)] \leq \frac{1}{c_r}.$$

hence

$$1-\frac{1}{c_r} \leq \mathbb{P}[Y \leq c_r \cdot \mathrm{ep}^{\mathcal{T}}(G,r)] = \frac{1}{N}\sum_{u\in S^{\mathcal{T}}}\big(\mathcal{T}(u)+\mathcal{L}(u)\big).$$

Fix $u \in S^{\mathcal{T}}$ and denote by $\mathrm{ep}_u = \mathrm{ep}(G,r,u)$ its extinction probability. By definition of $S^{\mathcal{T}}$ we have $\mathrm{ep}_u \leq c_r\cdot \mathrm{ep}^{\mathcal{T}}(G,r)$, and since G is an amplifier under temperature initialization, we have $\mathrm{ep}^{\mathcal{T}}(G,r) \leq 1/r$, therefore $\mathrm{ep}_u \leq \frac{r+1}{2r}$. Plugging this into the bound given by Lemma 2 we obtain

$$\begin{aligned}
\mathcal{T}(u)+\mathcal{L}(u) &\leq \frac{r\cdot \mathrm{ep}_u \cdot(1-\mathcal{L}(u))}{1-\mathrm{ep}_u} + \mathcal{L}(u) && \text{(by Lemma 2)}\\
&\leq \frac{r\cdot \frac{r+1}{2r}(1-\mathcal{L}(u))}{1-\frac{r+1}{2r}} + \mathcal{L}(u) && \text{(since it is increasing in } \mathrm{ep}_u)\\
&= \frac{r(r+1)}{r-1} - \mathcal{L}(u)\cdot \underbrace{\left(\frac{r(r+1)}{r-1}-1\right)}_{\geq 0 \text{ for any } r>1}\\
&\leq \frac{r(r+1)}{r-1}.
\end{aligned}$$

This yields

$$\frac{r-1}{r+1} = 1 - \frac{1}{c_r} \le \frac{1}{N} \sum_{u \in S^{\mathcal{T}}} \big(\mathcal{T}(u) + \mathcal{L}(u)\big) \le \frac{1}{N} \cdot |S^{\mathcal{T}}| \cdot \frac{r(r+1)}{r-1},$$

hence $|S^{\mathcal{T}}| \ge N \cdot \frac{(r-1)^2}{r(r+1)^2}$ as claimed.

□

We are now ready to prove Theorem A.

Theorem A. *Fix $r > 1$.*

1. *If G is an r-amplifier under uniform initialization then*

$$\mathrm{FT}(G,r) \ge \frac{(r-1)^2}{r^4(r+1)} \cdot \frac{1}{\mathrm{ep}(G,r)} \cdot \log\left(N \cdot \frac{r-1}{r+1} - 1\right) = \Omega\left(\frac{1}{\mathrm{ep}(G,r)} \cdot \log N\right).$$

2. *If G is an r-amplifier under temperature initialization then*

$$\mathrm{FT}^{\mathcal{T}}(G,r) \ge \frac{(r-1)^2}{r^4(r+1)} \cdot \frac{1}{\mathrm{ep}^{\mathcal{T}}(G,r)} \cdot \log\left(N \cdot \frac{(r-1)^2}{r(r+1)^2} - 1\right) = \Omega\left(\frac{1}{\mathrm{ep}^{\mathcal{T}}(G,r)} \cdot \log N\right).$$

Proof. Both claims are proved in essentially the same way.

1. Let $c_r = \frac{r+1}{2} > 1$ and consider the set

$$S = \{u \in V\colon\ \mathrm{ep}(G,r,u) \le c_r \cdot \mathrm{ep}(G,r)\}$$

of nodes with not too high an extinction probability ("cold nodes"). By Lemma 3 we have $|S| \ge N \cdot \frac{r-1}{r+1}$. Moreover, for any node $u \in S$ we have

$$\begin{aligned}
\mathcal{T}(u) &\le \frac{r \cdot \mathrm{ep}(G,r,u)}{1 - \mathrm{ep}(G,r,u)} && \text{(by Lemma 2)}\\
&\le \frac{r \cdot c_r \cdot \mathrm{ep}(G,r)}{1 - c_r \cdot \mathrm{ep}(G,r)} && \text{(since } u \in S)\\
&\le \frac{r \cdot \frac{r+1}{2} \cdot \mathrm{ep}(G,r)}{1 - \frac{r+1}{2} \cdot \frac{1}{r}} && \text{(since } \mathrm{ep}(G,r) \le 1/r)\\
&= \frac{r^2(r+1)}{r-1} \cdot \mathrm{ep}(G,r),
\end{aligned}$$

where throughout the computation all denominators are positive. Therefore we can apply Lemma 1 to all nodes $u \in V$ with $k = |S| - 1 = N \cdot \frac{r-1}{r+1} - 1$ and $t^\star = \frac{r^2(r+1)}{r-1} \cdot \mathrm{ep}(G,r)$ to get

$$\begin{aligned}
\mathrm{FT}(G,r) &= \frac{1}{N} \sum_{u \in V} \mathrm{FT}(G,r,u)\\
&\ge \frac{1}{N} \sum_{u \in V} \mathrm{fp}(G,r,u) \cdot \frac{\log k}{r \cdot t^\star} && \text{(by Lemma 1)}\\
&= \mathrm{fp}(G,r) \cdot \frac{\log k}{r \cdot t^\star}\\
&\ge \left(1 - \frac{1}{r}\right) \cdot \frac{\log(|S|-1)}{r} \cdot \frac{r-1}{r^2(r+1) \cdot \mathrm{ep}(G,r)} && \text{(since } \mathrm{fp}(G,r) \ge 1 - 1/r)\\
&= \frac{(r-1)^2}{r^4(r+1)} \cdot \frac{1}{\mathrm{ep}(G,r)} \cdot \log\left(N \cdot \frac{r-1}{r+1} - 1\right)\\
&= \Omega\left(\frac{1}{\mathrm{ep}(G,r)} \cdot \log N\right).
\end{aligned}$$

2. Likewise, let $c_r = \frac{r+1}{2} > 1$ and consider the set

$$S^{\mathcal{T}} = \{u \in V\colon\ \mathrm{ep}(G,r,u) \le c_r \cdot \mathrm{ep}^{\mathcal{T}}(G,r)\}$$

of nodes with not too high an extinction probability ("cold nodes"). By Lemma 3 we have $|S^{\mathcal{T}}| \ge N \cdot \frac{(r-1)^2}{r(r+1)^2}$. By exactly the same computation as before, for any node $u \in S$ we have

$$\begin{aligned}
\mathcal{T}(u) &\le \frac{r\cdot \mathrm{ep}(G,r,u)}{1-\mathrm{ep}(G,r,u)} && \text{(by Lemma 2)}\\
&\le \frac{r\cdot c_r\cdot \mathrm{ep}^{\mathcal{T}}(G,r)}{1-c_r\cdot \mathrm{ep}^{\mathcal{T}}(G,r)} && \text{(since } u\in S^{\mathcal{T}})\\
&\le \frac{r\cdot \frac{r+1}{2}\cdot \mathrm{ep}^{\mathcal{T}}(G,r)}{1-\frac{r+1}{2}\cdot\frac{1}{r}} && \text{(since } \mathrm{ep}^{\mathcal{T}}(G,r)\le 1/r)\\
&= \frac{r^2(r+1)}{r-1}\cdot \mathrm{ep}^{\mathcal{T}}(G,r),
\end{aligned}$$

where again all denominators are positive.

Therefore we can apply Lemma 1 to all nodes $u \in V$ with $k = |S^{\mathcal{T}}|-1 = N\cdot\frac{(r-1)^2}{r(r+1)^2}-1$ and $t^\star = \frac{r^2(r+1)}{r-1}\cdot \mathrm{ep}^{\mathcal{T}}(G,r)$ to get

$$\begin{aligned}
\mathrm{FT}^{\mathcal{T}}(G,r) &= \frac{1}{N}\sum_{u\in V}\big(\mathcal{T}(u)+\mathcal{L}(u)\big)\cdot \mathrm{FT}(G,r,u)\\
&\ge \frac{1}{N}\sum_{u\in V}\big(\mathcal{T}(u)+\mathcal{L}(u)\big)\cdot \mathrm{fp}(G,r,u)\cdot\frac{\log k}{r\cdot t^\star} && \text{(by Lemma 1)}\\
&= \mathrm{fp}^{\mathcal{T}}(G,r)\cdot \frac{\log k}{r\cdot t^\star}\\
&\ge \frac{(r-1)^2}{r^4(r+1)}\cdot\frac{1}{\mathrm{ep}^{\mathcal{T}}(G,r)}\cdot\log\left(N\cdot\frac{(r-1)^2}{r(r+1)^2}-1\right)\\
&= \Omega\left(\frac{1}{\mathrm{ep}^{\mathcal{T}}(G,r)}\cdot \log N\right). \qquad \square
\end{aligned}$$

3.2 Selection reactors are superamplifiers

The goal of this section is to prove Item 1 of Theorem B.

Let $S_{i,j}$ be a configuration consisting of $i \in [0,\ell]$ mutants among the leaves and $j \in [0,n]$ mutants in the hub.

The following lemma states that whenever the hub consists mostly of residents, we are at least a constant $r' = \frac{1}{2}(r+1) > 1$ more likely to gain a mutant there rather than to lose one. It holds since $p_{\mathrm{out}} = o(1)$ and $\ell\cdot p_{\mathrm{in}} = o(n)$.

Lemma 4. Consider the Moran process starting from a configuration $S_{i,j}$ with $1 \le j \le n/2$. Let $S_{i',j'}$ be the first subsequent configuration with $j' \ne j$. Then, for all large enough ℓ, we have

$$\frac{\mathbb{P}[j'=j+1]}{\mathbb{P}[j'=j-1]} \ge \frac{1}{2}(r+1) > 1.$$

Proof. Let $p^+(i,j)$ (resp. $p^-(i,j)$) be the probability that when in configuration $S_{i,j}$, the number j of mutants in the hub increases (resp. decreases) in a single step. Let $\mathsf{F} = \mathsf{F}(S_{i,j}) = N + (r-1)(i+j)$ be the total fitness. Straightforward computation gives

$$p^+(i,j) \ge \frac{rj}{\mathsf{F}}\cdot(1-p_{\mathrm{out}})\cdot\frac{n-j}{n}$$

and

$$p^-(i,j) \le \underbrace{\frac{n-j}{\mathsf{F}}\cdot(1-p_{\mathrm{out}})\cdot\frac{j}{n}}_{A} + \underbrace{\frac{\ell}{\mathsf{F}}\cdot p_{\mathrm{in}}\cdot\frac{j}{n}}_{B}$$

where in $p^+(i,j)$ we counted only the "within-hub" interactions and in $p^-(i,j)$ we bounded by the worst case when all leaf nodes are residents.

We claim that $B = o(A)$: Indeed, we have $n - j \geq \frac{1}{2}n$ and $1 - p_{\text{out}} = 1 - 1/\alpha(N)^2 = \Theta(1)$, hence the claim is equivalent with $\ell p_{\text{in}} = o(n)$ which is true in our case as $\ell p_{\text{in}} = \ell/\alpha(N)^2$ and $n = \ell/\alpha(N)$. Therefore,

$$\lim_{N\to\infty} \frac{p^+(i,j)}{p^-(i,j)} \geq \lim_{N\to\infty} \frac{\frac{rj}{\mathsf{F}} \cdot (1 - p_{\text{out}}) \cdot \frac{n-j}{n}}{\frac{n-j}{\mathsf{F}} \cdot (1 - p_{\text{out}}) \cdot \frac{j}{n}} = r.$$

In particular, for all large enough N the ratio is higher than a smaller positive constant $\frac{1}{2}(r+1)$ and thus also

$$\frac{\mathbb{P}[j' = j+1]}{\mathbb{P}[j' = j-1]} \geq \min_i \frac{p^+(i,j)}{p^-(i,j)} \geq \frac{1}{2}(r+1). \qquad \square$$

The next lemma states that, with high probability, the first mutant appears in a leaf node. It holds since $n = o(\ell)$ and $p_{\text{in}} = o(1)$.

Lemma 5. Under both uniform and temperature initialization, the first mutant is initialized at a leaf node with high probability.

Proof. This is straightforward computation. For uniform initialization, the first mutant appears at a leaf with probability $\ell/N \to_{N\to\infty} 1$.

Under temperature initialization, any leaf node u satisfies $\mathcal{L}(u) + \mathcal{T}(u) \geq \mathcal{L}(u) = 1 - p_{\text{in}} \to_{N\to\infty} 1$, hence the overall fixation probability is at least $\ell/N \to_{N\to\infty} 1$ too. $\square$

The next lemma states that, with high probability, the initial mutant produces a mutant subpopulation in the hub of a superconstant size. It holds since $h = \omega(1)$ and $h = o(n^3)$.

Lemma 6. Consider the Moran process starting from a configuration $S_{i,j}$ with $i \geq 1$. Then, with high probability, the process reaches a configuration $S_{i',j'}$ with $j' \geq h^{1/3}$.

Proof. Fix a leaf u that hosts a mutant in the initial configuration $S_{i,j}$.

First, we claim that, with high probability, u fires in the hub $\sqrt{h}$ times before being replaced by a resident: The probability p^+ that in a single step u is selected for reproduction and fires in the hub satisfies $p^+ \geq \frac{1}{N} \cdot p_{\text{in}}$. The probability p^- that in a single step u is replaced by a resident firing out from the hub satisfies $p^- \leq \frac{n}{N} \cdot p_{\text{out}} \cdot \frac{1}{\ell}$. Hence as long as u contains a mutant, the probability that u fires in the hub before being replaced by a resident satisfies

$$p = \frac{p^+}{p^+ + p^-} \geq \frac{\frac{\ell p_{\text{in}}}{n p_{\text{out}}}}{1 + \frac{\ell p_{\text{in}}}{n p_{\text{out}}}} = \frac{h}{1+h} > 1 - \frac{1}{h}.$$

The probability q that u fires in the hub at least $\sqrt{h}$ times before being replaced satisfies $q \geq p^{\sqrt{h}} = (1 - 1/h)^{\sqrt{h}}$. Since $h = \omega(1)$, by Proposition 3 this event indeed happens with high probability as claimed.

Next, we claim that after $\sqrt{h}$ mutant fire-ins, the hub contains at least $h^{1/3}$ mutants, with high probability. Note that this claim is not obvious, for at least two reasons: First, if the hub was biased towards losing mutants (which it is not), it could happen that each invading mutant gets swiftly eliminated. Second, if the hub was very small (e.g. a constant size), it could happen that the fire-ins from u repeatedly replace the same node.

To prove the second claim, run the process until either the hub contains at least $h^{1/3}$ mutants, or u has fired in the hub $\sqrt{h}$ times. Let t be the (random, stopping) time when the earlier of those two conditions occurs and let p_t be the probability that the configuration $S_{i',j'}$ at time t still satisfies $j' < h^{1/3}$. Consider the potential $\phi(S_{i,j}) = j$ that counts the number of mutants in the hub. As in the proof of Lemma 4, when $j \leq h^{1/3} \leq n/2$, the hub together with all the resident fire-ins is still biased towards gaining mutants (or not biased either way when $j = 0$). Hence in steps when u does not fire in, the potential ϕ does not decrease in expectation. Moreover, in steps when u does fire in, the potential increases in expectation by at least $(n-j)/n \geq 1 - h^{1/3}/n = 1 - o(1)$, where the last equality holds due to $h = o(n^3)$. Hence

$$h^{1/3} \geq \mathbb{E}[\phi(S_{i',j'})] = (1-p_t) \cdot h^{1/3} + p_t \cdot \left(\underbrace{\phi(S_{i,j})}_{\geq 0} + \underbrace{\sqrt{h} \cdot (1 - o(1))}_{=\omega(h^{1/3})} \right) \geq (1-p_t) \cdot h^{1/3} + p_t \cdot \omega(h^{1/3}),$$

thus $p_t = o(1)$ as desired. $\square$

The next lemma states that once the hub contains a superconstant number of mutants, mutants eventually gain majority in the hub, with high probability. It holds since $h = \omega(1)$ and $n = \omega(1)$.

Lemma 7. Let $S_{i,j}$ be a configuration with $j \geq h^{1/3}$. Then, with high probability, the process reaches a configuration $S_{i',j'}$ with $j' \geq n/2$.

Proof. By Lemma 4, for any $j \in [1, n/2]$ the mutant subpopulation in the hub is a constant $r' = \frac{1}{2}(r+1)$ more likely to increase rather than decrease. Hence the probability p that, starting with j mutants, the mutant subpopulation in the hub increases to size $n/2$ before being eliminated, can be lower-bounded by the absorption probability of a 1-dimensional random walk with a constant forward bias r' as

$$p \geq \frac{1 - 1/(r')^j}{1 - 1/(r')^{n/2}}.$$

Since both j and $n/2$ are $\omega(1)$, we have

$$\lim_{N\to\infty} \frac{1 - 1/(r')^j}{1 - 1/(r')^{n/2}} = \frac{1}{1} = 1. \qquad \square$$

The next lemma states that once the mutants have majority in the hub, fixation on the whole graph occurs with high probability. It holds since $h = \omega(1)$ and $h = o(n)$.

Lemma 8. Let $S_{i,j}$ be a configuration with $j \geq n/2$. Then, with high probability, the process reaches the configuration $S_{\ell,n}$.

Proof. We will show that, in fact, once we have $j = \omega(h)$ mutants in the hub, fixation on the whole graph occurs with high probability (this suffices since $h = o(n)$). For any configuration $S_{i,j}$, define its "star-like" potential by

$$\phi(S_{i,j}) = \beta^i \cdot \gamma^j,$$

where $\beta = \frac{h+r}{hr^2+r} \to \frac{1}{r^2}$ and $\gamma = \frac{hr+1}{hr+r^2} \to 1$ are positive real numbers less than one. In particular, we have $\phi(S_{0,0}) = 1$ and $\phi(S_{\ell,n}) \to_{N\to\infty} 0$. Also, since $j = \omega(h)$ we have $\phi(S_{i,j}) \leq \gamma^j = (1 - \Theta(1/h))^{\omega(h)} \to_{N\to\infty} 0$. As we verify below, the potential is chosen such that it does not change in expectation when the reproduction event happens along an edge that connects a hub node with a leaf node. Moreover, for reproduction events that happen along edges within the hub, the potential decreases in expectation. Thus the function ϕ is a sub-martingale and by Doob's optional stopping theorem, the expectation $\phi^\infty = \mathbb{E}[\phi(S_T)]$ of the potential at the (random, stopping) time T when the process terminates satisfies

$$\phi(S_{i,j}) \geq \phi^\infty = \mathrm{fp}_{i,j} \cdot \phi(S_{\ell,n}) + (1 - \mathrm{fp}_{i,j}) \cdot \phi(S_{0,0}),$$

where $\mathrm{fp}_{i,j}$ is the fixation probability starting from the configuration $S_{i,j}$. Rearranging the terms and dividing by the (positive) expression $\phi(S_{0,0}) - \phi(S_{\ell,n})$, this gives

$$\mathrm{fp}_{i,j} \geq \frac{\phi(S_{0,0}) - \phi(S_{i,j})}{\phi(S_{0,0}) - \phi(S_{\ell,n})} \to_{N\to\infty} \frac{1-0}{1-0} = 1.$$

To verify our claim, it suffices to check contributions of three different types of edges:

(a) "within-hub" edges connecting a mutant and a resident: When this edge is used for reproduction, it is r times more likely that we gain a mutant rather than lose it. Denoting the current potential by x, we need to show that

$$\frac{r}{r+1} x \cdot \gamma + \frac{1}{r+1} x \cdot \frac{1}{\gamma} \leq x.$$

This reduces to $(1-\gamma)(r\gamma - 1) \geq 0$ which is true because the first term is positive and the second one is positive for any large enough population size (recall that $h = \omega(1)$).

(b) "resident leaf – mutant hub" edge: The edge is used in the direction towards the hub with probability $p_1 = \frac{1}{\mathsf{F}} \cdot p_{\text{in}} \cdot \frac{1}{n}$ and in the direction towards the leaf with probability $p_2 = \frac{r}{\mathsf{F}} \cdot p_{\text{out}} \cdot \frac{1}{\ell}$. Hence we need to verify that

$$\frac{p_1}{p_1 + p_2} \cdot x \cdot \frac{1}{\gamma} + \frac{p_2}{p_1 + p_2} \cdot x \cdot \beta = x.$$

This reduces to showing

$$\frac{h}{h+r} \cdot \frac{1}{\gamma} + \frac{r}{h+r} \cdot \beta = 1$$

which is true since the left-hand side rewrites as

$$\frac{h}{h+r}\cdot\frac{hr+r^2}{hr+1}+\frac{r}{h+r}\cdot\frac{h+r}{hr^2+r}=\frac{hr(h+r)+(h+r)}{(h+r)(hr+1)}=1.$$

(c) "mutant leaf – resident hub" edge: Similarly as in the previous case, we get $p_1=\frac{r}{\mathsf{F}}\cdot p_{\text{in}}\cdot\frac{1}{n}$ and $p_2=\frac{1}{\mathsf{F}}\cdot p_{\text{out}}\cdot\frac{1}{\ell}$. Then we need to verify

$$\frac{hr}{hr+1}\cdot\gamma+\frac{1}{hr+1}\cdot\frac{1}{\beta}=1$$

which is done by rewriting the left-hand side as

$$\frac{hr}{hr+1}\cdot\frac{hr+1}{hr+r^2}+\frac{1}{hr+1}\cdot\frac{hr^2+r}{h+r}=\frac{h}{h+r}+\frac{r}{h+r}=1.$$ □

We are now ready to prove Item 1 of Theorem B.

Theorem B (Reactors are fast strong amplifiers)**.** *For any function* $\alpha\colon\mathbb{N}\to\mathbb{R}$ *that is both* $o(N^{1/2})$ *and* $\omega(1)$*, and for any fixed* $r>1$*, the selection reactors* $\{\mathrm{SR}^\alpha\}_{N=1}^{\infty}$ *satisfy the following, under both uniform and temperature initialization:*

1. *(strong amplifiers)* $\mathrm{fp}(\mathrm{SR}^\alpha_N,r)\to_{N\to\infty}1$.
2. *(fast)* $\mathrm{FT}(\mathrm{SR}^\alpha_N,r)=\mathcal{O}(\log N\cdot\alpha^5(N))$.

Proof of Theorem B, Item 1. Each of the following four steps happens with high probability: By Lemma 5, the first mutant appears at a leaf node. From there, by Lemma 6, this mutant establishes a mutant subpopulation in the hub with superconstant size $j\geq h^{1/3}$. From there, by Lemma 7, the mutant subpopulation in the hub grows until the mutants have majority in the hub. From there, by Lemma 8, the mutants fixate. □

3.3 Selection reactors are fast superamplifiers

The goal of this section is to prove Item 2 of Theorem B.

Denote the configuration with i mutant leaf-nodes and j mutant hub-nodes by $S_{i,j}$. Let $h_0=\lfloor h\rfloor$ be the parameter $h=(\ell\cdot p_{\text{in}})/(n\cdot p_{\text{out}})$ rounded down to the nearest integer. We define $\psi(S_{i,j})=h_0\cdot i+j$. Intuitively, each mutant leaf-node is worth h_0, each mutant hub-node is worth 1, and the potential of a configuration is the total worth of its mutant nodes. The potential function ψ is a rescaled and rounded version of the function $\psi'(S)=\sum_{v\in S}1/\deg(v)$ which was used in several earlier works such as [7, 8, 9].

First we show that, in expectation, each active step increases the potential by at least a constant c (that depends on $r>1$). The claim is essentially the same as Lemma 2 in [8] and Lemma 67 in [9].

Lemma 9. Fix a selection reactor $\mathrm{SR}(\ell,n,p_{\text{in}},p_{\text{out}})$ and $r>1$. Fix a configuration S different from $S_{0,0}$ and $S_{\ell,n}$. Sample a random active step of the process and denote the new configuration by $S'\neq S$. Let

$$c_1=\frac{r-1}{r+1},$$
$$c_2=r-1-\frac{r^2}{h+r}=r-1-\mathcal{O}(1/h),$$
$$c_2=\frac{r-1}{r}-\frac{r-1}{rh+1}=\frac{r-1}{r}-\mathcal{O}(1/h),$$

be positive constants and let $c=\min\{c_1,c_2,c_3\}$. Then

$$\mathbb{E}[\psi(S')\mid S]\geq\psi(S)+c.$$

Proof. We say that an edge is *active* if its two endpoints are occupied by one mutant and one resident. There are three types of active edges:

1. *Type 1 edges* ("hub-hub") that connect a mutant in the hub to a resident also in the hub;
2. *Type 2 edges* ("hub-leaf") that connect a mutant in the hub to a resident in a leaf; and

3. *Type 3 edges* ("leaf-hub") that connect a mutant in a leaf to a resident in the hub.

Each active edge can be used during a replacement event in either of its two directions. Fix an active edge uv with u a mutant and v a resident and suppose it has type i (where $1 \le i \le 3$). Below we show that, assuming the edge uv is selected for replacement (in one of its two directions), the potential increases at least by c_i in expectation. Since in any one step of the active process, some active edge is used for replacement, we conclude that each active step increases the potential by at least $\min_i\{c_i\} = c$, in expectation.

We treat the three types of edges separately. For two nodes x, y let $p_{x\to y}$ be the probability that in a single step of the Moran process, node x replaces node y. Let $\mathsf{F} = \ell + n + (i+j)(r-1)$ be the total fitness of the population at a configuration $S_{i,j}$.

1. Type 1 edges ("hub-hub"): If the mutant at a hub-node u replaces a resident at hub-node v, the potential increases by 1, otherwise it decreases by 1. We have

$$p_{u\to v} = \frac{rj}{\mathsf{F}} \cdot (1 - p_{\text{out}}) \cdot \frac{n-j}{n} \quad \text{and} \quad p_{v\to u} = \frac{n-j}{\mathsf{F}} \cdot (1 - p_{\text{out}}) \cdot \frac{j}{n},$$

thus, in expectation, the potential increases by

$$\frac{p_{u\to v} \cdot (+1) + p_{v\to u} \cdot (-1)}{p_{u\to v} + p_{v\to u}} = \frac{r-1}{r+1} = c_1.$$

2. Type 2 edges ("hub-leaf"): If the mutant at a hub-node u replaces a resident at leaf-node v, the potential increases by h_0, otherwise it decreases by 1. We have

$$p_{u\to v} = \frac{rj}{\mathsf{F}} \cdot p_{\text{out}} \cdot \frac{\ell-i}{\ell} \quad \text{and} \quad p_{v\to u} = \frac{\ell-i}{\mathsf{F}} \cdot p_{\text{in}} \cdot \frac{j}{n},$$

thus, in expectation, the potential increases by

$$\frac{p_{u\to v} \cdot (+h_0) + p_{v\to u} \cdot (-1)}{p_{u\to v} + p_{v\to u}} = \frac{h_0 \cdot r - h}{h+r} \ge \frac{(h-1)r - h}{h+r} = r - 1 - \frac{r^2}{h+r} = c_2$$

3. Type 3 edges ("leaf-hub"): If the mutant at a leaf-node u replaces a resident at hub-node v, the potential increases by 1, otherwise it decreases by h_0. We have

$$p_{u\to v} = \frac{ri}{\mathsf{F}} \cdot p_{\text{in}} \cdot \frac{n-j}{n} \quad \text{and} \quad p_{v\to u} = \frac{n-j}{\mathsf{F}} \cdot p_{\text{out}} \cdot \frac{i}{\ell},$$

thus, in expectation, the potential increases by

$$\frac{p_{u\to v} \cdot (+1) + p_{v\to u} \cdot (-h_0)}{p_{u\to v} + p_{v\to u}} = \frac{hr - h_0}{hr+1} \ge \frac{hr - h}{hr+1} = \frac{r-1}{r} - \frac{r-1}{hr+1} = c_3.$$

In particular, for any fixed $r > 1$ and as $N \to \infty$ we have $h \to \infty$ and thus $c = c_1 = (r-1)/(r+1)$ is a constant that only depends on r. ☐

Let R_k be the expected number of times the active process attains a potential value k. The next lemma bounds R_k from above.

Lemma 10. Fix an integer k. Run the active process starting from any configuration. Then the expected total number of visits to configurations T that satisfy $\psi(T) = k$ is $\mathcal{O}(h^2)$.

Proof. Run the active process until the potential value k is attained for the first time (if it is never attained, the claim is trivial). Denote the corresponding configuration by S_0 and for $t \ge 0$ let $\mathcal{S}_t$ be a random variable denoting the configuration after t active steps, starting from S_0 ($\mathcal{S}_0 = S_0$). Consider a function $\xi(\mathcal{S}_t) = \psi(\mathcal{S}_t) - c \cdot t$, where c is the positive constant from Lemma 9. We claim that the function ξ is a sub-martingale with differences bounded by $h_0 + c$: Indeed, since

$$\begin{aligned}
\mathbb{E}[\xi(\mathcal{S}_{t+1}) \mid \mathcal{S}_t] &= \mathbb{E}[\psi(\mathcal{S}_{t+1}) - c \cdot (t+1) \mid \mathcal{S}_t] \\
&= \mathbb{E}[\psi(\mathcal{S}_{t+1}) \mid \mathcal{S}_t] - c \cdot (t+1) \\
&\ge \psi(\mathcal{S}_t) + c - c \cdot (t+1) && \text{(by Lemma 9)}, \\
&= \psi(\mathcal{S}_t) - c \cdot t = \xi(\mathcal{S}_t),
\end{aligned}$$

the sequence is a sub-martingale and since

$$|\xi(\mathcal{S}_{t+1}) - \xi(\mathcal{S}_t)| = |\psi(\mathcal{S}_{t+1}) - \psi(\mathcal{S}_t) - c| \le |\psi(\mathcal{S}_{t+1}) - \psi(\mathcal{S}_t)| + c \le h_0 + c,$$

its differences are upper-bounded by $h_0 + c$.

By Azuma's inequality [10] we can bound the probability that after precisely t steps the potential is at most its initial value as

$$\mathbb{P}[\psi(\mathcal{S}_t) \le \psi(S_0)] = \mathbb{P}[\xi(\mathcal{S}_t) - \xi(\mathcal{S}_0) \le -ct] \le \exp\left(\frac{-c^2 t^2}{2t \cdot (c+h_0)^2}\right) \le \left(e^{\frac{-c^2}{2(c+h_0)^2}}\right)^t.$$

Summing the infinite geometric series over $t = 0, \ldots, \infty$ we obtain an upper bound on the expected number of visits to states T with $\phi(T) \le \phi(S_0)$, and thereby also on the number of visits to states with $\phi(T) = \phi(S_0)$. Specifically, we denote the exponent by $x = \frac{-c^2}{2(c+h_0)^2}$ and note that since $x \in (-1/2, 0)$, we have $e^x \le 1 + x/2$. Upon summing the series we obtain the desired

$$\sum_{t=0}^{\infty} (e^x)^t = \frac{1}{1-e^x} \le \frac{1}{1-(1+x/2)} = -\frac{2}{x} = \frac{4\,(c+h_0)^2}{c^2} = \mathcal{O}(h^2).$$ □

Next we move our attention to the waiting process. Given a configuration $S_{i,j}$, let $p_{i,j}$ be the probability that the next step of the process is active. The number $W_{i,j}$ of waiting steps the process makes when at $S_{i,j}$ before we observe an active steps then satisfies $W_{i,j} = 1/p_{i,j}$. The next lemma establishes a lower bound on $p_{i,j}$.

Lemma 11. Consider a selection reactor with $n \le \ell$ and any configuration $S = S_{i,j}$ different from $S_{0,0}$ and $S_{\ell,n}$. Then the probability $p_{i,j}$ that the next step of the process is active satisfies

$$p_{i,j} \ge \frac{p_{\text{in}}}{2r\ell \cdot n} \cdot \left(i(n-j) + (\ell - i)j\right).$$

Proof. We account for the fire-in events only. Let $\mathsf{F} = \ell + n + (r-1)(i+j)$ be the total fitness. Since $n \le \ell$ we have $\mathsf{F} \le 2r\ell$. The probability p_r that a resident fires in the hub and replaces a mutant equals

$$p_r = \frac{\ell - i}{\mathsf{F}} \cdot p_{\text{in}} \cdot \frac{j}{n} = \frac{p_{\text{in}}}{\mathsf{F} \cdot n} \cdot (\ell - i)j$$

and the probability p_m that a mutant fires in the hub and replaces a resident equals

$$p_m = \frac{ri}{\mathsf{F}} \cdot p_{\text{in}} \cdot \frac{n-j}{n} \ge \frac{p_{\text{in}}}{\mathsf{F} \cdot n} \cdot i(n-j)$$

Hence $p_{i,j} \ge p_r + p_m \ge \frac{p_{\text{in}}}{2r\ell \cdot n} \cdot (i(n-j) + (\ell - i)j)$ as desired. □

Next, for each $k \in [1, \ell \cdot h_0 + n - 1]$ let $W_k = \max\{W_{i,j} \mid \psi(S_{i,j}) = k\}$ be the largest possible number of waiting steps we can expect to encounter, when at a configuration with potential value equal to $\psi(S_{i,j}) = k$. The following lemma bounds the sum $\sum_{k=1}^{\ell h_0 + n - 1} W_k$ from above.

Lemma 12. Suppose $n \le \ell/4$. Then

$$\sum_{k=1}^{\ell h_0 + n - 1} W_k = \mathcal{O}\left(N \log N \cdot \frac{h}{p_{\text{in}}}\right).$$

Proof. Split the configuration space into subsets where each subset X_k contains configurations $S_{i,j}$ with $k = \psi(S_{i,j}) = h_0 \cdot i + j$. First, within each slice, we identify the configuration where the bound on $p_{i,j}$ from Lemma 11 is weakest. To that end, it suffices to analyze the expression

$$t(i,j) = i(n-j) + (\ell - i)j$$

for a fixed $k = h_0 \cdot i + j$. Note that $t(i,j) = t(\ell - i, n - j)$.

Plugging in $j = k - h_0 \cdot i$ we get

$$t(i,j) = i(n - (k - h_0 i)) + (\ell - i)(k - h_0 i) = 2h_0 \cdot i^2 - i \cdot (2k + h_0 \ell - n) + k\ell$$

which is a quadratic function of i with a positive coefficient $2h_0$ by the leading term. Its minimum over real numbers is attained for $i_0 = (2k + h_0\ell - n)/4h_0$.

Note that when $k \le \frac{1}{2}(\ell h_0 + n) - n$ then we have $i_0 \ge k/h_0$ which implies that the corresponding j_0 satisfies $j_0 \le 0$, thus the minimum of $t(i, j)$ over real numbers is attained at a point outside of the allowed configuration space. For such k we thus get

$$t(i, j) \ge t(k/h_0, 0) = \frac{n}{h_0} \cdot k.$$

Similarly, when $k \ge \frac{1}{2}(\ell h_0 + n) + n$ we have $i_0 \le (k - n)/h_0$ and the corresponding j_0 satisfies $j_0 \ge n$. For such k we thus get

$$t(i, j) \ge t((k - n)/h_0, n) = \frac{n}{h_0} \cdot (\ell h_0 + n - k).$$

Finally, for the $2n + 1$ intermediate values $k \in [\frac{1}{2}(\ell h_0 + n) - n, \frac{1}{2}(\ell h_0 + n) + n]$, note that any corresponding configuration $S_{i,j}$ satisfies $i \in [\frac{1}{2}\ell - \frac{3n}{2h_0}, \frac{1}{2}\ell + \frac{3n}{2h_0}]$. Since $t(i, j)$ is non-decreasing in j when $i \le \frac{1}{2}\ell$ and non-increasing in j when $i \ge \frac{1}{2}\ell$, together with the symmetry $t(i, j) = t(\ell - i, n - j)$ this implies that

$$t(i, j) \ge t\left(\frac{1}{2}\ell - \frac{3n}{2h_0}, 0\right) \ge t\left(\frac{1}{8}\ell, 0\right) = \frac{1}{8}\ell n,$$

where for convenience in the second inequality we used a bound $n \le \ell/4$ which holds for all large enough ℓ.

It remains to compute the sum over all $k \in [1, \ell h_0 + n - 1]$. Using Lemma 11, the above analysis and the fact that $h_0 = o(\ell)$ we can write

$$\begin{aligned}
\sum_{k=1}^{\ell h_0+n-1} W_k &\le \frac{2r\ell n}{p_{\text{in}}} \cdot \left(\frac{h_0}{n} \cdot \sum_{k=1}^{\frac{1}{2}(\ell h_0+n)-n} \frac{1}{k} \;+\; \frac{h_0}{n} \cdot \sum_{k=\frac{1}{2}(\ell h_0+n)+n}^{\ell h_0+n-1} \frac{1}{\ell h_0 + n - k} \;+\; (2n+1) \cdot \frac{8}{\ell n} \right) \\
&\le \frac{2r\ell}{p_{\text{in}}} \cdot (h_0 \cdot \log(\ell h_0) + h_0 \cdot \log(\ell h_0) + 5) \\
&= \mathcal{O}\left(\frac{\ell}{p_{\text{in}}} \cdot h_0 \log \ell\right) = \mathcal{O}\left(N \log N \cdot \frac{h}{p_{\text{in}}}\right). \qquad \square
\end{aligned}$$

We are now ready to prove Item 2 of Theorem B.

Theorem B (Reactors are fast strong amplifiers). *For any function $\alpha\colon \mathbb{N} \to \mathbb{R}$ that is both $o(N^{1/2})$ and $\omega(1)$, and for any fixed $r > 1$, the selection reactors $\{\mathrm{SR}^\alpha\}_{N=1}^\infty$ satisfy the following, under both uniform and temperature initialization:*

1. *(strong amplifiers)* $\mathrm{fp}(\mathrm{SR}^\alpha_N, r) \to_{N\to\infty} 1$.
2. *(fast)* $\mathrm{FT}(\mathrm{SR}^\alpha_N, r) = \mathcal{O}(\log N \cdot \alpha^5(N))$.

Proof of Theorem B, Item 2. For each $k \in [1, \ell h_0 + n - 1]$, consider the "slice" X_k consisting of configurations $S_{i,j}$ with $k = \psi(S_{i,j}) = h_0 \cdot i + j$. Let R_k be the expected number of times a slice X_k is visited (by an active step) and let W_k be an upper bound on the expected number of steps before the slice is left, once it is visited. Rescaling from steps to generations we then have

$$\mathrm{FT}(\mathrm{SR}^\alpha_N, r) \le \frac{1}{N} \sum_{k=1}^{\ell h_0+n-1} R_k \cdot W_k.$$

By Lemma 10, for any k we have $R_k = \mathcal{O}(h^2)$. Hence by Lemma 12 we have

$$\mathrm{FT}(\mathrm{SR}^\alpha_N, r) = \mathcal{O}(h^2) \cdot \frac{1}{N} \cdot \sum_{k=1}^{\ell h_0+n-1} W_k = \mathcal{O}(\log N \cdot h^3/p_{\text{in}}).$$

Since we have $h = \Theta(\alpha(N))$ and $p_{\text{in}} = 1/\alpha^2(N)$, this gives the desired bound $\mathcal{O}(\log N \cdot \alpha^5(N))$. $\square$

References

[1] Ewens, W. J. *Mathematical population genetics 1: theoretical introduction*, vol. 27 (Springer Science & Business Media, 2012).

[2] Moran, P. A. P. *et al.* The statistical processes of evolutionary theory. *The statistical processes of evolutionary theory.* (1962).

[3] Tkadlec, J., Pavlogiannis, A., Chatterjee, K. & Nowak, M. A. Population structure determines the tradeoff between fixation probability and fixation time. *Communications Biology* **2**, 138 (2019). URL `https://doi.org/10.1038/s42003-019-0373-y`.

[4] Cormen, T. H. *Introduction to algorithms* (MIT press, 2009).

[5] Goldberg, L. A. *et al.* Asymptotically optimal amplifiers for the moran process. *Theoretical Computer Science* **758**, 73–93 (2019).

[6] Flajolet, P., Gardy, D. & Thimonier, L. Birthday paradox, coupon collectors, caching algorithms and self-organizing search. *Discrete Applied Mathematics* **39**, 207–229 (1992).

[7] Díaz, J. *et al.* Approximating fixation probabilities in the generalized moran process. *Algorithmica* **69**, 78–91 (2014).

[8] Chatterjee, K., Ibsen-Jensen, R. & Nowak, M. A. Faster monte-carlo algorithms for fixation probability of the moran process on undirected graphs. *arXiv preprint arXiv:1706.06931* (2017).

[9] Ann Goldberg, L., Lapinskas, J. & Richerby, D. Phase transitions of the moran process and algorithmic consequences. *Random Structures & Algorithms* **56**, 597–647 (2020).

[10] Azuma, K. Weighted sums of certain dependent random variables. *Tohoku Mathematical Journal, Second Series* **19**, 357–367 (1967).
